# Les luxations bilatérales antérieures pures des épaules à mécanisme particulier: à propos de deux cas

**DOI:** 10.11604/pamj.2015.22.178.7435

**Published:** 2015-10-22

**Authors:** Kaldadak Koufagued, Bouchaib Chafry, Salim Bouabid, Belkacem Chagar

**Affiliations:** 1 Service de Traumatologie Orthopédie 2, Faculté de Médecine et de Pharmacie de Rabat, Hôpital Militaire d'instruction Mohammed V, Rabat, Maroc

**Keywords:** Epaule, luxation antérieure, bilatérale pure, mécanisme particulier, shoulder, anterior dislocation, pure bilateral, special mechanism

## Abstract

Les luxations bilatérales antérieures pures des épaules sont des entités cliniques rares. Une trentaine de cas sont décrits dans la littérature. Le mécanisme varie d'un cas à l'autre, les épaules en abduction et en rétro-pulsion, coudes en extention et en supination a été décrite une seule fois dans la littérature. A ce propos, les auteurs rapportent ce même mécanisme particulier de luxation antérieure bilatérale pure des épaules chez deux jeunes patients, et discutent des circonstances, mécanisme de survenue, du traitement et du pronostique.

## Introduction

Les luxations gléno-humérales sont les plus fréquentes de toutes les luxations, la forme bilatérale est rare, dominée par la variante postérieure qui survient souvent dans le cadre du «triple E syndrome» [1]. Les auteurs rapportent deux nouveaux cas de luxation antérieure pure, à mécanisme particulier rapporté pour la seconde fois et discutent des divers mécanismes, du traitement et du pronostique.

## Patient et observation

### Observation ^°^ 1

**Figure 1:**
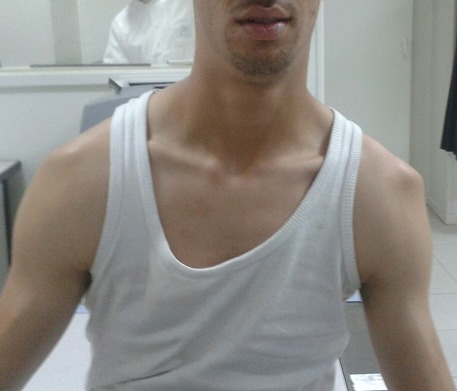
Aspect clinique de la luxation bilatérale des épaules chez le premier patient

**Figure 2:**
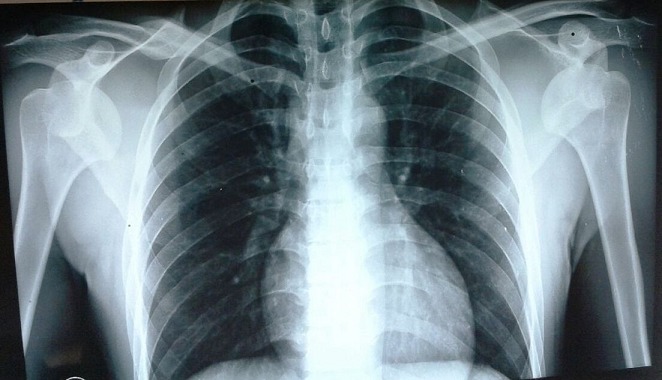
Radiographie des épaules de face montrant la Luxation antéro-interne dans sa variété sous-coracoïdienne chez le premier patient

**Figure 3:**
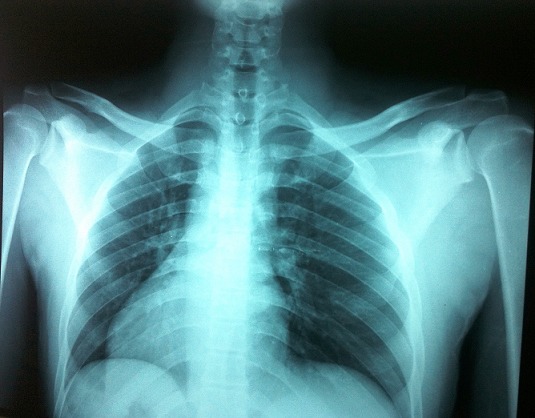
Radiographie des épaules de face après réduction montrant les deux têtes humérales en place

**Figure 4:**
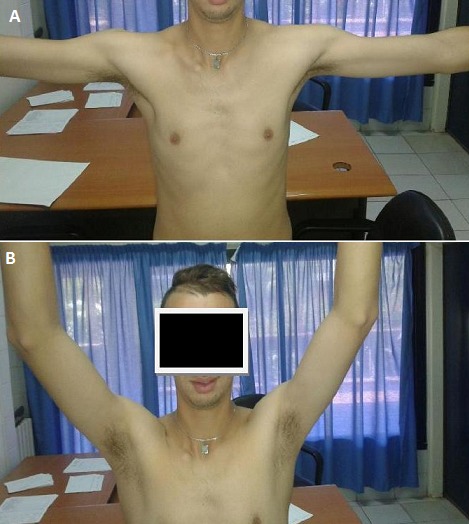
A) et B): Aspect clinique à 9 semaines post-traumatique chez le premier patient

Il s'agit d'un patient de 23 ans sportif, sans antécédents pathologiques notables, reçu aux urgences pour traumatisme fermé des deux épaules suite à un accident de la voie publique (AVP); motocycliste heurté en arrière par une voiture; entrainant une chute avec réception sur les deux mains projetées en arrière, épaules en abduction et rétro-pulsion, coudes en extension et en supination; provoquant une douleur intense et une impotence fonctionnelle totale des deux épaules. L'examen clinique, symétrique et bilatéral, retrouve les signes de luxation antérieure bilatérale des épaules (Figure 1). La sensibilité et la motricité dans le territoire du nerf axillaire étaient conservées, les pouls radiaux présents. La radiographie conventionnelle des épaules a confirmé le diagnostic de luxations bilatérales pures des épaules dans leur variété antérieure sous-coracoïdienne (Figure 2). Sous anesthésie générale, les luxations ont été réduites par la manœuvre de Milch puis la stabilité testée (Figure 3 A et B). Les deux épaules ont été immobilisées en adduction et rotation interne par des orthèses pendant trois semaines. Le patient a bénéficié à l'issue de séances de rééducation fonctionnelle. A neuf semaines de l'accident, la mobilité des deux épaules était très satisfaisante (Figure 4) avec des amplitudes articulaires de 160^°^ en abduction, 40^°^ en rotation externe et la rotation interne atteint D4 de façon bilatérale. Au dernier recul à 9 mois du traumatisme, les épaules étaient stables sans récidive ni installation d'instabilité.

### Observation n^°^ 2

**Figure 5:**
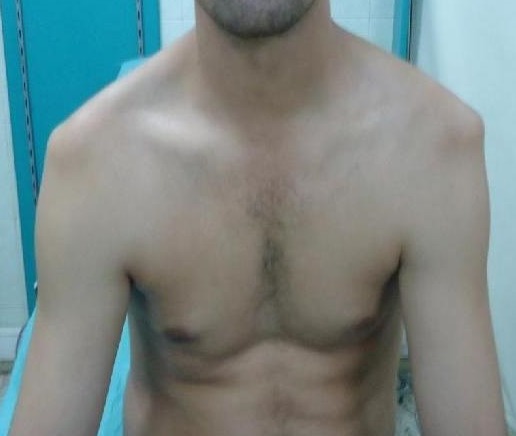
Aspect clinique de la luxation bilatérale des épaules chez le deuxième patient

**Figure 6:**
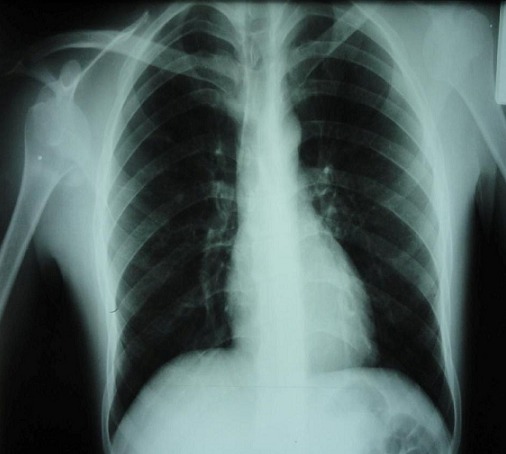
Radiographie des épaules de face montrant la Luxation antéro-interne dans sa variété sous-coracoïdienne chez le deuxième patient

**Figure 7:**
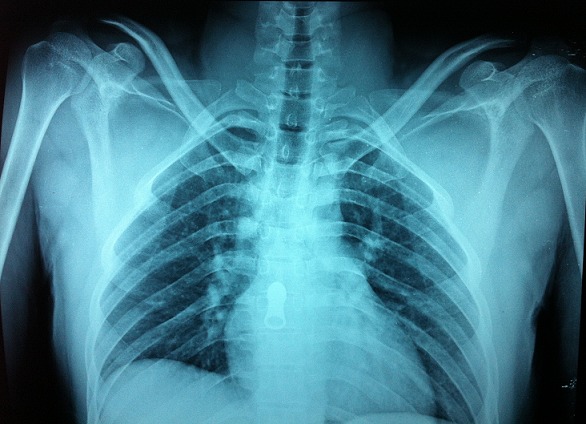
Radiographie des épaules de face montrant les deux têtes humérales en place chez le deuxième patient à trois semaines du traumatisme

Il s'agit d'un patient de 35 ans, sans antécédents pathologiques notables, reçu aux urgences pour traumatisme fermé des deux épaules suite à une chute d'une hauteur d'environ deux mètres. Le patient rapporte qu'il était sur une échelle qui a glissé en avant le propulsant en arrière entrainant une chute avec réception sur les 2 mains, les épaules en abduction et en retro-pulsion; coudes en extension et en supination entrainant une douleur intense et une impotence fonctionnelle totale des deux épaules. À l'examen clinique, les signes de luxation antérieure bilatérale étaient présents (Figure 5). La sensibilité et la motricité dans le territoire des nerfs axillaires étaient conservées et le pouls radial bilatéral était présent. La radiographie conventionnelle des épaules a confirmé le diagnostic de luxations pures des deux épaules dans leur variété antérieure sous-coracoïdienne (Figure 6). Sous anesthésie générale et par la manœuvre de Milch les deux luxations ont été réduites, avec testing de la stabilité. Les deux épaules ont été immobilisées en adduction et rotation interne par des orthèses pendant trois semaines puis s'en est suivie la rééducation fonctionnelle (Figure 7). A neuf semaines de l'accident, les amplitudes articulaires des deux épaules étaient de 160^°^ en abduction à droite et 150^°^ à gauche, 40^°^ en rotation externe à droite et 35^°^ à gauche et la rotation interne atteint D4 de façon bilatérale. On n'a noté ni récidive, ni instabilité au dernier recul à 21 mois du traumatisme.

## Discussion

La luxation bilatérale des épaules a été décrite pour la première fois en 1902 suite à des contractions musculaires excessives provoquées par un surdosage au camphre [2]. Elles ont fait l'objet de peu de publications [3, 4]. Brown [5] en 1984 a individualisé, sur une série de 90 cas de luxations bilatérales, trois différentes étiologies: les violentes contractions musculaires (49%), les traumatismes directs (23%) et l'absence de tout traumatisme (36%). Ces luxations peuvent être postérieures; variété la plus fréquente [6, 7]; décrites par Brackstone [1] sous le nom de «triple E syndrome» (l’épilepsie ou toute crise convulsive, l’électrocution et l'extrême traumatisme); antérieures rares avec seulement une trentaine de cas rapportés [6] ou inférieures qui en représentent 0,5% [7–10]. Les circonstances et les mécanismes de survenue de la luxation antérieure bilatérale chez notre premier patient étaient un accident de la voie publique, ayant entrainé une chute en arrière avec réception sur les deux mains, épaules en abduction et rétro-pulsion; coudes en extension et en supination. La chute aurait donc accentué l'abduction et la rotation externe des deux épaules à l'origine de la luxation antérieure bilatérale. Pour notre deuxième cas, les circonstances et le mécanisme étaient un accident de travail, chute d'une échelle d'environ deux mètres avec réception du patient sur les deux mains et épaules en abduction et en retro-pulsion, coudes en extension supination. Les circonstances de survenue des luxations étaient différentes mais le mécanisme était identique chez nos deux patients. En faisant la revue de la littérature, Abalo et Al. [11] rapportent une observation d'un patient de 37 ans, victime d'un accident de la voie publique (AVP), (arrière d'une moto heurté par une voiture) ayant entrainé une chute avec réception sur les deux mains épaule en abduction et retro-pulsion; coude en extension (Figure 1). L'AVP et le mécanisme sont identiques à ceux de notre premier patient sauf que pour le cas d'Abalo et Al, la luxation bilatérale était associée à une fracture parcellaire de la grosse tubérosité de l'humérus à droite alors qu'elle était bilatérale pure chez notre patient. Suite à ce mécanisme, il s'en suit le passage de la tête humérale à travers la faiblesse anatomique entre le ligament gléno-huméral moyen et inférieur ou foramen de Rouvière à l'origine de la luxation antéro-interne sous coracoïdienne [12].

Nous rapportons donc ce même mécanisme pour la seconde fois et chez deux patients. D'autres mécanismes inhabituels ont été décrits. Singh et Kumar [13] ont rapporté un cas ou les deux épaules ont été luxées par des mécanismes différents, chez un patient présentant des antécédents d'instabilité de l’épaule droite. La luxation gauche antérieure était post traumatique secondaire à une chute de moto avec réception directe sur l’épaule alors que le côté droit a été luxé secondairement en antérieur lors du transport, patient tenu par le membre supérieur droit. Bouras et al. [6] ont décrit un cas de luxation antérieure bilatérale des épaules chez un jeune bodybuilder de dix huit ans qui lors d'une séance de musculation, alors qu'il soulevait une barre droite de 40 kg, celle-ci a basculé en arrière provoquant la luxation. Les étiologies de luxations bilatérales antérieures rapportées sont tous post traumatique alors qu'elles sont non traumatiques dans les luxations postérieures et dues souvent à des contractions musculaires excessives. Le traitement chez nos deux patients a été orthopédique par une réduction des luxations sous anesthésie générale pour lutter contre la douleur, le stress, l'anxiété et ne pas traumatiser le patient et d'entrainer des lésions supplémentaire par la technique de Milch qui consiste à placer le bras en abduction à 150^°^, en repoussant la tête humérale d'une main, puis à ramener le membre en rotation interne coude au corps. De nombreuses techniques de réduction des luxations sont décrites [14] mais nous adoptons cette dernière car c'est la technique de réduction que nous maîtrisons le plus et elle nous donne entière satisfaction. Quelque soit la manœuvre de réduction utilisée, elle doit être douce et progressive afin de ne pas aggraver les lésions [15]. Le caractère bilatéral des luxations n'a pas changé la prise en charge d'autant plus qu'elle est réalisé sous anesthésié général avec un bon relâchement musculaire. La chirurgie n’étant envisagée qu'en cas de récidive; bien que celle-ci soit plus fréquente chez les patients âgés de moins de 40 ans [15]. Nos deux patients bien qu'ayant moins de 40 n'ont pas eu de récidive au dernier recul. Le pronostic est bon après une bonne rééducation fonctionnelle.

## Conclusion

L’étiologie post traumatique des luxations bilatérales antérieures pure des épaules sont exceptionnelles. Nous rapportons pour la deuxième fois le caractère inhabituel du mécanisme causal par ces deux cas cliniques.
